# Longitudinal Study-Based Dementia Prediction for Public Health

**DOI:** 10.3390/ijerph14090983

**Published:** 2017-08-30

**Authors:** HeeChel Kim, Hong-Woo Chun, Seonho Kim, Byoung-Youl Coh, Oh-Jin Kwon, Yeong-Ho Moon

**Affiliations:** 1 Science and Technology Management Policy, University of Science & Technology, Daejeon 34113, Korea; kimhc@ust.ac.kr; 2 Korea Institute of Science and Technology Information, Seoul 02456, Korea; seonho@kist.re.kr (S.K.); cohby@kisti.re.kr (B.-Y.C.); dbajin@kisti.re.kr (O.-J.K.); 3 Convergence Research Center for Diagnosis, Treatment and Care System of Dementia, Korea Institute of Science and Technology, Seoul 02792, Korea; 4 Science and Technology Information Science, University of Science & Technology, Daejeon 34113, Korea

**Keywords:** public health, aging, dementia, big data, machine learning, support vector machine

## Abstract

The issue of public health in Korea has attracted significant attention given the aging of the country’s population, which has created many types of social problems. The approach proposed in this article aims to address dementia, one of the most significant symptoms of aging and a public health care issue in Korea. The Korean National Health Insurance Service Senior Cohort Database contains personal medical data of every citizen in Korea. There are many different medical history patterns between individuals with dementia and normal controls. The approach used in this study involved examination of personal medical history features from personal disease history, sociodemographic data, and personal health examinations to develop a prediction model. The prediction model used a support-vector machine learning technique to perform a 10-fold cross-validation analysis. The experimental results demonstrated promising performance (80.9% F-measure). The proposed approach supported the significant influence of personal medical history features during an optimal observation period. It is anticipated that a biomedical “big data”-based disease prediction model may assist the diagnosis of any disease more correctly.

## 1. Introduction

Research investigating the treatment of dementia has been performed worldwide for more than 20 years; however, no curative therapy has been established [1]. Dementia is one of the most predominant diseases, and the number of affected patients has increased rapidly in the elderly population globally. According to the World Alzheimer Report published in 2015, the number of individuals with dementia is expected to increase from 44 million in 2013 to more than 135 million in 2050 [2]. From a public health perspective, Korea has been affected by the aging of its population. From 2000 to 2014, the rate of change has been 1.5 times faster than that of Japan, and five times faster than that of France [3]. This trend of rapid aging creates public health problems, including increased medical expenses and a decrease in active roles for the elderly, who have experienced a deepening sense of alienation [4]. The rapidly aging population of Korea has led to a rapid increase in the number of individuals affected by dementia. According to a prevalence survey conducted by the Ministry of Health and Welfare, the number of dementia patients in Korea was approximately 540,000 in 2012, which is projected to increase rapidly to 840,000 in 2020 [5]. In other words, Korea’s dementia population is the fastest growing such group in the world, and the resulting public health cost caused by dementia is expected to reach 1.5% of gross domestic product in 2050 [6].

Although there are approved medicines for dementia treatment, including donepezil, rivastigmine, galantamine, *para*-aminobenzoic acid, coumarin, flavonoids, and pyrrolo-isoxazole, among others, they have no effect on the fundamental treatment of dementia, but only delay the progression of the disease [1]. In 2011, the World Alzheimer Report reported that these medicines were relatively more effective when applied in the early stages of dementia [7]. Therefore, early diagnosis by prediction of dementia plays a crucial role in symptom relief of dementia. Symptom relief through early prediction is expected to reduce public health costs [8]. Thus, predicting the onset of dementia is an urgent public health issue, and would mitigate sudden increases in the disease and the burden on public health care costs [6].

Many investigators have been conducting research on predicting diseases using “big data” analysis techniques. To predict disease, The National Health Service of the United Kingdom collected and analyzed prescription data from pharmacies and hospitals nationwide [9]. The United States National Institutes of Health has a project known as “Pillbox”, in which big data are used through the National Library of Medicine [10]. Johns Hopkins University (Baltimore, MD, USA) developed a disease prediction system using the social media service *Twitter* [11]. The Seton Healthcare Family (Austin, TX, USA) and IBM Joint Development Program have analyzed and tracked medical information, and have predicted outcomes of two million patients per year [12]. Researchers have also been active in pursuing studies that use big data of medical history in Korea. Most notably, the Korea National Health Insurance Service Senior Cohort (KNHIS-SC) database (DB) was created to help treat geriatric diseases and, based on this DB, Hwang et al. found that calcium-channel blockers had a protective effect on the risk for dementia among elderly Koreans with hypertension [13].

The proposed research aimed to early predict dementia and improve an individual’s diagnostic performance, and used a biomedical big data approach that included personal disease history, sociodemographic data, and personal health examination, referred to herein as “personal medical history”. The KNHIS-SC DB contains information regarding participant insurance eligibility, medical treatment, and general health examination data for all Koreans [14]. Several machine learning (ML) techniques have been applied to incorporate useful features from the data in the DB. Section 2 presents several previous studies, and the methodology is explained in Section 3. Section 3 contains the introduction of KNHIS-SC DB with several statistics, feature selection, and approaches to support the effectiveness of personal medical history. Our experimental results, the discussion, and conclusion are described in Section 4, Section 5 and Section 6, respectively.

## 2. Previous Work 

The work of many researchers in traditional studies investigating dementia have revealed the impact of medical history that includes sociodemographic and lifestyle data, medical records, and biophysical properties [15,16,17,18,19,20]. Based on medical history, many researchers have performed studies to predict dementia using big data techniques. Some studies have attempted to use medical records, and sociodemographic and lifestyle data [21,22,23]. Others have attempted to predict Alzheimer’s disease through genomic analysis, brain imaging, and medical record review [24,25,26]. Still others have attempted to predict dementia by changing the examination battery for dementia and its biophysical properties [27,28,29].

Shim et al. [21] investigated the development of mild cognitive impairment to full-blown dementia by observing 778 Korean patients; predictions of risk factors were made through Cox proportional hazard models. The results indicated that age, APOE and high CDR-SOB score, are risk factors. Walters et al. [22] conducted a dementia prediction analysis using data from 930,395 individuals 60 to 95 years of age in the United Kingdom. They developed an algorithm for a prediction model by combining cohort sociodemographic, cardiovascular and lifestyle data, and mental health variables collected over a period of five years. The advantage of this model is that it can be developed through routinely collected data. 

Allen et al. [24] conducted a competition for the prediction of Alzheimer’s disease, in which 527 research teams from around the world participated. A total of 2069 studies were submitted. One of the leading studies was published by Zhu et al. [25], who optimized ML techniques to predict mild cognitive impairment and Alzheimer’s disease (a type of dementia) in a pool of 489 residents of the United States. Alzheimer’s Disease Neuroimaging Initiative data and genomic data were used to predict changes in mini-mental state examination (MMSE) score and, as a result, Alzheimer’s disease was predicted with a probability of 69%. Stephan et al. [26] conducted a dementia prediction study using data from 1721 individuals 65 years of age and older in three cities in France. Patients, who were examined using magnetic resonance imaging (MRI), had been observed for a 10 years period. The authors investigated whether it was useful to include MRI imaging in conventional dementia predictors such as age, poor neuropsychological test performance, subjective memory complaints, low educational attainment, sex, depression, cardiovascular, etc. 

Stefano et al. [27] investigated cognitive decline over a five-year period in 1435 elderly individuals 70 years of age or older in the GuidAge Prevention trial in France. The McNair & Kahn Scale and Visual Analogue Scales measured patient’s memory function and impairment in everyday life. They performed various neuropsychological tests such as MMSE, CDRsB, FCSRT, Trail Making Test, categorical and lexical verbal fluencies, Instrumental Activities of Daily Living and Geriatric Depression Scale. Nowrangi et al. [28] predicted dementia from data collected from 7625 volunteers at the National Alzheimer Coordinating Center in the United States. They found that behavioral factors, such as filing taxes, remembering dates, and significant travel affected prediction of dementia progression by surveying patients using the Functional Activities Questionnaire (FAQ).

Dementia prediction in individuals of the United States and Europe has been a relatively popular research topic compared with that of Asian-based studies. The proposed approach in the present article aims to predict dementia in a representative Korean population using KNHIS data. Because all medical records of Koreans have been automatically collected by KNHIS under a national policy, the KNHIS DB is a genuinely representative source. The proposed approach analyzed sociodemographic, health examination, and personal disease history data in the KNHIS-SC DB to determine the most salient features and the optimal observation period for dementia prediction.

## 3. Longitudinal Study-Based Dementia Prediction

### 3.1. Workflow

**Figure 1:**
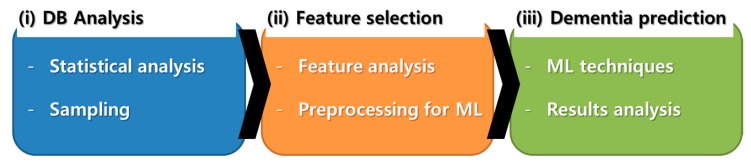
Dementia prediction process. DB, database; ML, machine learning.

To predict dementia, the proposed approach uses personal medical history including sociodemographic, health examination history, and personal disease history. Figure 1 illustrates the dementia prediction process: analysis of the KNHIS-SC DB and extraction of samples for experiments; feature selection and preprocessing for applying ML techniques; prediction model optimization with the best combination of features.

### 3.2. Introduction of KNHIS-SC DB

**Figure 2:**
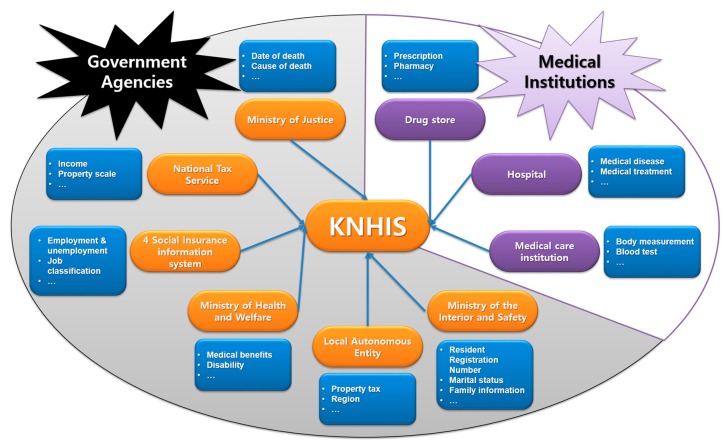
Personal medical data collection of Korea National Health Insurance Service (KNHIS) cooperating with other institutions.

Since 1989, the KNHIS has been a legal institution of the Korean medical insurance program, and has managed all health care costs for health care recipients, providers and government agencies in the Republic of Korea. The KNHIS can collect medical records of every citizen in Korea through every registered hospital, drug store, and medical care institution. Moreover, all personal medical history data is linked with other databases of government agencies. Thus, personal, family, and socioeconomic information can be used through the network, as shown in Figure 2.

By establishing the Korea National Health Information DB, the KNHIS has been able to centrally manage all medical records, including personal, sociodemographic, and medical treatment data [30]. Certified individuals or organizations can use the KNHIS DB.

The KNHIS-SC DB consists of 558,147 seniors, representing 10% of all Korean population, consisting of 5.5 million seniors. Population statistics for 5.5 million seniors of 2013 was compared with that of all Korean population to confirm the KNHIS-SC DB can be considered as a representative sample of Korean population. Statistics Korea (KOSTAT) calculate Korean population statistics based on resident registration every month [31] and we used the KOSTAT’s data of December 2013 as the information of Korean population. The comparison results supported that the KNHIS-SC DB can be considered as a representative sample of Korean population (Table 1).

**Figure 3:**
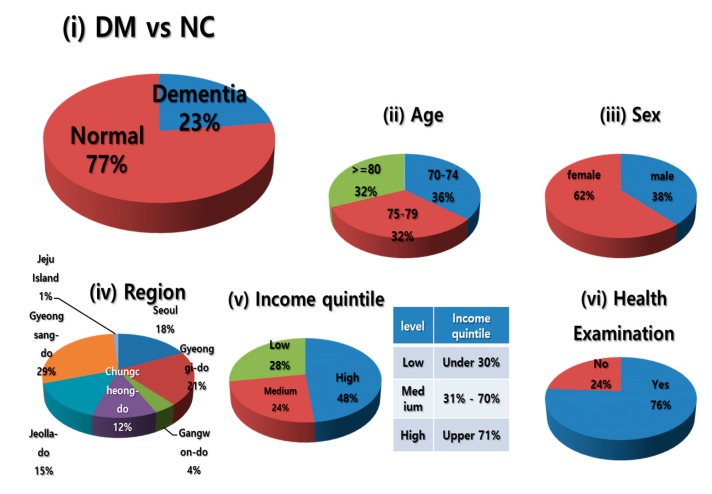
Distribution of the Korea National Health Insurance Service Senior Cohort (KNHIS-SC) database, as of 2013. DM, dementia group; NC, normal controls.

Table 2 summarizes the composition of the KNHIS-SC DB population according to age and year. Data from 2002 to 2013 for the population that was 60 years of age or older as of 2002, were generated to form a total of 672 levels according to sex, region, and income quintile. Figure 3 illustrates the composition ratio according to the following criteria: (i) dementia group (DM) vs. normal control group (NC), (ii) age, (iii) sex, (iv) region, (v) income quintile and (vi) previous health examination history.

Table 3 describes the content of the KNHIS-SC DB. The KNHIS-SC DB includes data on insurance eligibility, income, medical service benefits, medical records, long-term care, and health examination history. The participant insurance eligibility DB (PIE-DB) includes demographic data, socioeconomic level and other data. The medical treatment DB (MT-DB) includes treatment items and treatment disease data and the general health examination DB (GHE-DB) includes the medical examination history, ranging from physical measurement to past medical record. In addition, the Medical Care Institution DB (MCI-DB) contains data such as the type, area and establishment period of the medical care institution, the number of beds in the institution, the number of doctors, and equipment availability status. Finally, the Long-term Care Insurance DB (LCI-DB) includes long-term care application and decision results, doctors′ opinions such as a recognized needs survey, and data regarding the status of long-term care facilities. The KNHIS-SC DB provides the reliable data structure and a variety of variables for the sample. Because of these characteristics, studies investigating geriatric diseases have been conducted using the KNHIS-SC DB [13,32].

### 3.3. Feature Selection

#### 3.3.1. Feature Analysis

Personal medical history, including sociodemographic data, lifestyle, personal disease history, biophysical properties, and other factors have been widely used to predict dementia. The proposed approach selected those items as features for applying ML techniques. Among the items in the KNHIS-SC DB, the proposed approach selected: (i) sociodemographic data (e.g., sex, age, income quintile) in PIE-DB. In addition, (i) body measurement data (e.g., height, weight, body mass index, waist, blood pressure highest, blood pressure lowest); (ii) blood test results (e.g., blood glucose level before meals and levels of total cholesterol, hemoglobin, serum GOT, serum GPT, and gamma-GTP); (iii) urinary test results; (iv) history of personal illness (e.g., stroke, heart disease, high blood pressure, diabetes, hyperlipidemia, phthisis, cancer); (v) history of family illness (e.g., stroke, heart disease, high blood pressure, diabetes, cancer); and (vi) smoking status in GHE-DB; and finally (vii) personal disease history in MT-DB.

Personal disease history of MT-DB is composed of three-characters of approximately 2600 *International Classification of Diseases-10* (ICD-10) codes [33]. The ICD-10 code consists of one alphabetic character (from A to Z, primary disease groups), and two digits from 00 to 99 (extended disease groups). The ICD-10, a standard classification of diagnosis for the purpose of epidemiology study and health management, is popularly used by medical doctors of all WHO member states. Members of WHO use ICD-10 to classify medical records and obtain statistical data and other health status information. WHO provides ICD-10 standard manuals in 46 languages. However, it is possible to assign different codes based on medical doctors. The ICD-10 is used to monitor the overall health status of the population, and to monitor the incidence and prevalence of diseases, and other health problems in relation to various individual variables such as personal characteristics and environment. The three-character categories of the ICD-10 code are constructed using the following two criteria: (1) single disease or disease group: classified according to the frequency, severity, sensitivity to public health interventions of the disease or with respect to common characteristics; and (2) other: rare diseases with different characteristics [34]. According to personal disease history recorded in the KNHIS-SC DB, the rate of the disease corresponding to (1) was 78.4% and the rate of (2) was 21.6%. This result shows that the number of diagnoses is relatively small, although the range of diseases included in (2) may be wide.

#### 3.3.2. Preprocessing

In the preprocessing step, the selected medical history features are processed into feature types suitable for ML. In PIE-DB, sex was divided into male and female. After that, age was used as seven levels and income quintile was classified as three levels. In MT-DB, the proposed approach verified personal disease history with their ICD-10 codes. Finally, in GHE-DB, the height of 101–230 cm was classified into 13 levels in 10 cm increments and the weight of 26–300 kg was classified into 11 levels in 5 kg increments. Waist, body mass index, blood test and urine test were classified as normal and abnormal according to the criteria of the health examination standards (Ministry of Health and Welfare Notice No. 2016-11). Table 4 shows the normal/abnormal criterial range for GHE-DB items [35]. History of personal/family illness distinguishes existence and non-existence.

Each feature was categorized in every year from 2003 to 2013 to identify time-series patterns. In particular, feature items of GHE-DB measured change between the corresponding year and 2013. Trends of increase/decrease and normal/abnormal change compared with 2013 were featured.

Instances are classified by DM and NC based on whether the corresponding instance was diagnosed with dementia based on MT-DB diagnosis data during 2013. The government of Korea supports medical expenses on dementia patients diagnosed by the following ICD-10 codes: “Dementia in Alzheimer disease” (F00, G30), “Vascular dementia” (F01), “Dementia in other diseases classified elsewhere (Dementia with Lewy bodies, Creutzfeldt-Jakob disease, and Dementia in human immunodeficiency virus [HIV] disease are included)” (F02), and “Unspecified dementia” (F03). The proposed approach used the same criteria to classify dementia [36,37].

### 3.4. Dementia Prediction Using Longitudinal Public Health Data

The proposed approach aimed to prove the following two hypotheses: (1) personal medical history has an effect on improving the performance of dementia prediction; and (2) personal disease history is the most important information in the medical history. To prove the first hypothesis, the proposed approach used personal medical history from 2003 to 2013. PIE-DB, MT-DB and GHE-DB include various personal medical information. To compare the performance of the medical history, baseline experiments used only information from 2013. Furthermore, the proposed approach tried to determine the best period for predicting dementia. To find out the best period, the proposed approach set the period as the last three, five, seven, nine and 11 years from 2013, and analyzed changes between values of the corresponding year and those of 2013. Not only increase/decrease of values but also changes among classes were considered. The classes were normal/abnormal or predefined levels. To show the effectiveness of personal disease history in the second hypothesis, features from personal disease history were compared with other features and tried to make the best combination of features. 

In addition, the proposed approach examined to confirm the predictive power for the known dementia-related diseases and to reveal the effect of the other diseases to prediction. Table 5 summarizes previous studies that have revealed dementia-related diseases [38]. The proposed approach focuses on the patterns of personal disease history that can be biomarkers for dementia. Not only one disease history but also a diseases group can be a biomarker. Personal disease history in MT-DB was used to analyze such biomarkers. To overcome problems with data sparseness, the proposed approach used primary disease groups in the first experiment.

## 4. Experimental Results

### 4.1. Sampling

**Figure 4:**
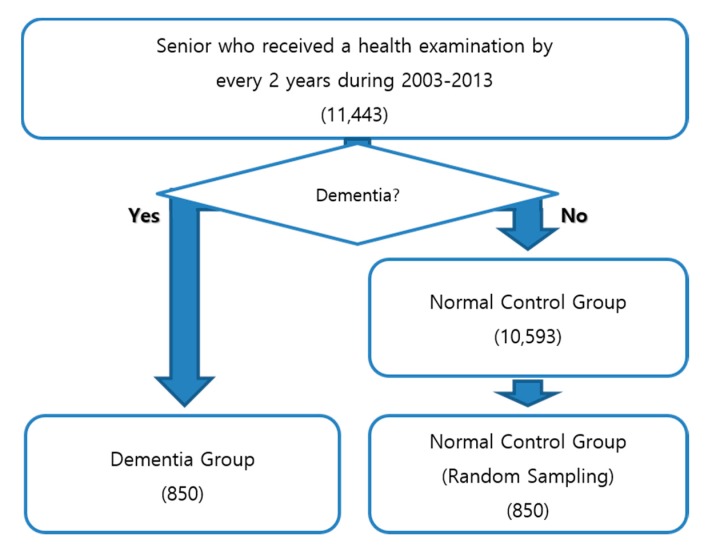
Data sampling process.

Because the number of instances was insufficient to apply ML techniques, the proposed approach followed some heuristic rules for sampling: (i) KNHIS provide free health examination for individuals older than 65 years of age, for every two years and the proposed approach used the health examination results from 2003 to 2013; (ii) after step (i), 11,443 instances remained and they consisted of 850 DMs and 10,593 NC; (iii) 850 NC and 850 DM were randomly selected and used in the experiments. Figure 4 summarizes the data sampling process.

### 4.2. Experimental Setting

We explored appropriate ML techniques for the prediction of DM and performed optimization to derive a DM prediction model. From the KNHIS-SC DB, we selected 850 seniors with dementia and 850 seniors without dementia. As for the features, four types of features were selected from the PIE-DB, 70 types from the GHE-DB, and, 2600 types from the MT-DB.

Table 6 describes features of the baseline experiment. To demonstrate the effectiveness of the time-series information, we used only features from 2013 in the baseline experiment.

The proposed approach used changes between values of the corresponding year and those of 2013 as well as features in the corresponding year. To find out the best period for predicting dementia, we set the periods as the last three, five, seven, nine and 11 years. Table 7 describes features that used in the longitudinal data-based experiments.

The proposed approach builds a dementia prediction model focused on personal medical history, and two experiments were conducted to determine the best method to use personal disease history and the optimal personal medical history period. First, longitudinal model 1 used the primary disease groups for personal disease history and longitudinal model 2 used the extended disease groups for personal disease history. Through the above experiments, we determined that the best method was to use personal disease history. To prove the effectiveness of personal medical history, we set a baseline experiment that used features of only one year, 2013. In addition, the above experiments tried to compare periods to determine the optimal personal medical history period to predict dementia.

### 4.3. Evaluation 

Since the proposed approach did not focus on algorithmic analysis of ML techniques, we focused on an analysis of feature combinations to show the best performance. Therefore, WEKA, which can easily compare and analyze the influence of features through the existing algorithm, was used. WEKA is the most useful for academic purposes because it contains most of the existing algorithms and most of the functions required for data mining from feature selection to model evaluation [39]. First, features are selected based on gain ratio attribute evaluation. Subsequently, we used support vector machine (SVM), which is one of the ML methods provided by WEKA. The algorithm used was Weka.classifiers.functions.SMO, which is one of the SVM provided by WEKA, the calibrator was logistic and the kernel was RBFKernel (C = 1.0, E = 1.0). Using the k-fold cross validation method, we verified the model with the 10-fold cross-validation method. The results measurements were evaluated using precision, recall and F-measure [40].

### 4.4. Results

The longitudinal features reflected the time-series changes in the baseline features over the years 2003–2012. The results of the longitudinal model 1 and baseline feature models are shown in Table 8. The Baseline results were 69.0% F-measure and 1.3%p–4.1%p increased F-measure when longitudinal features were added to baseline. The 2009–2013 model showed the best performance, with 73.1% F-measure. Longitudinal model 1 used PT-DB, GHE-DB, and MT-DB with the primary disease group.

In the next experiments, longitudinal model 2 used the extended disease group instead of the primary disease group (ex. Primary disease group E extended to E00, E01…E98, E99; each a primary disease group extended to 100 codes of extended disease group). Table 9 describes the second experimental results. To optimize the proposed approach, we extracted the relative influence of the features using the gain ratio attribute evaluation method, and features of relatively high influence were sequentially assembled. After that, all combinations of features have been used and finally the best combination was detected (Table 9). In longitudinal model 2, the 2007–2013 model was the best combination that of features consists of 409 features and demonstrated as F-measure of 80.9%.

Table 10 describes the best combination of features derived from longitudinal model 2, and contains five attributes related with blood tests in GHE-DB and 75 in the extended disease group with respect to MT-DB. Features of the primary disease groups F and G contain known dementia-related diseases. Additionally, features of the primary disease group M include newly detected dementia-related diseases through the proposed approach. Features of the primary disease group S and I indicate diseases for surgery with anesthesia and diseases for the circulatory system, respectively. So, these groups appear not to be directly related with dementia. However, many previous investigations reported that anesthetic experience or diseases of circulatory system affects dementia [41,42]. Moreover, features of the primary disease groups E, N, and R include both known and newly detected dementia-related diseases. As for the newly detected dementia-related diseases, they are needed to prove relationships with dementia through biological experiments. In addition to the disease features, GHE features also have an impact on predicting dementia. The blood test results of total cholesterol, hemoglobin, serum GOT, serum GPT and gamma GTP are features of dementia prediction from the GHE-DB.

## 5. Discussion

The current approach had several issues and limitations.

(1) KNHIS-SC DB is a database of records created by doctors’ medical service activities. The diagnosis rate of dementia in Korea is 73.6% (as of 2015) [43], which is higher than in some countries of in Europe (44–67%) [44]. As for the dementia diagnosis rate, the proposed approach does not appear to demonstrate a good performance. The proposed approach used a gold standard data in Korea.
(2) The proposed approach considered the NC based on only a diagnostic history of dementia. However, the elderly may have symptoms or diseases related with dementia. Such cases have ambiguous attributes to classify DM and NC, and may have a negative effect on the performance of dementia prediction. Therefore, the proposed approach involved an extra experiment excluding NC who had dementia-related symptoms or diseases in their medical history. The experimental result showed 81.4% F-measure (89.3 precision and 74.8 recall). Compared with the previous experiments (including NC who have dementia-related symptoms or diseases), dementia prediction slightly increased performance (+0.5% F-measure). This was because the attributes of NC became clearly different from DM.
(3) Since the current approach used the personal history of dementia, the results may be biased. However, the approach tried to follow the same method with the same data with human physicians. As for the reference, the experiment excluding dementia features demonstrated 73.3% accuracy, 77.2% precision, 66.1% recall, and 71.2% F-measure.
(4) Since the data from the KNHIS-SC DB in the proposed approach are newly released, there has been no previous work to handle it. To compare with the proposed method and the previous methods, the baseline experiment used only data from a particular year, and other experiments used personal medical history to prove the effectiveness of personal medical history.
(5) The proposed approach dealt with personal medical history according to year, i.e., if a person has a medical record for such a disease at least one time during the corresponding year, the proposed approach considered him or her as having that disease in one total year. Thus, the current method to deal with personal medical history is not sophisticated and it needs to handle personal medical history by the month, week, or day for a more specific dementia prediction.
(6) In addition, the proposed approach did not consider disease phase, but considered whether a person had certain diseases or not. To analyze disease patterns more exquisitely, we will develop and apply an algorithm that can allocate the phase for any diseases based on drug dosage and dose schedule screening.

## 6. Conclusions

A dementia prediction model for all of Korea was derived using the KNHIS-SC DB and ML techniques. Various features were analyzed and optimized to improve dementia prediction performance. Several experiments supported the effectiveness of personal medical history with promising performance. This study was the first attempt to construct a dementia prediction model based on a representative sample of the Korean population; this is especially significant because the proposed approach demonstrated state-of-the-art performance (i.e., 80.9% F-measure). The results confirm the possibility of dementia prediction using personal medical history data. Longitudinal models 1 and 2 showed that a seven-year period and a three-year period were optimal observation periods. Relatively recent information was more effective in predicting dementia. In other words, a longer observation period did not result in better performance.

The results demonstrate that the personal disease history of the personal medical history has an important role in dementia prediction. Five attributes related to blood tests of personal health examination and 58 diseases included in personal disease history have been extensively studied, individually or in part, in association with dementia in previous studies. The proposed approach confirmed the same results with the previous research based on a representative cohort DB housing information about the entire Korean population. Furthermore, 18 newly detected diseases were considered to be associated with dementia in personal disease history. Because dementia is strongly related to aging, mental illness, and head injury, the diseases listed in Table 10 may not be the only meaningful features that predict dementia. Future research topics will include determining meaningful diseases related only to dementia. 

Although the proposed approach focuses on improving performance of individual dementia diagnoses, the experimental results may contribute to a reduction in the incidence of dementia, not only in Korea, but around the world.

## Figures and Tables

**Table 1:** Comparison of KNHIS-SC DB and Resident registration population construct ration according to sex, age, and region (as of 2013).

Item	Classification	KNHIS-SC DB (%)	Population Statistics Based on Resident Registration (%)
**Sex**	Male	38	39
	Female	62	61
	Sum	100	100
**Age**	70–74	36	42
	75–79	32	30
	80+	32	28
	Sum	100	100
**Region**	Seoul	18	18
	Gyeonggi-do	21	23
	Gyeongsang-do	29	28
	Jeolla-do	15	14
	Gangwon-do	4	4
	Chungcheong-do	12	12
	Jeju Island	1	1
	Sum	100	100

**Table 2:** KNHIS-SC DB population.

Year	Total	Age, Years					
		60–64	65–69	70–74	75–79	80–85	85+
2002	558,147	196,116	147,361	97,657	61,217	35,215	20,581
2003	557,195	161,261	157,155	105,339	66,978	41,351	25,111
2004	539,278	122,144	164,629	111,768	71,595	42,769	26,373
2005	521,967	85,776	167,293	120,415	76,092	44,336	28,055
2006	504,417	46,113	173,449	128,909	80,206	45,795	29,945
2007	487,460		186,057	135,631	84,926	47,983	32,863
2008	470,005		151,331	142,687	89,766	50,578	35,643
2009	452,631		114,559	149,864	96,030	54,763	37,415
2010	436,395		80,560	153,026	104,029	58,905	39,875
2011	422,171		43,571	160,221	112,412	63,101	42,866
2012	405,614			172,606	118,931	67,529	46,548
2013	388,493			140,503	125,795	71,993	50,202

**Table 3:** KNHIS-SC DB contents.

**PIE-DB**	Demographic information (sex, age, area of residence)
	Death related information (date of death, cause of death)
	Types of health insurance (health insurance subscribers/medical benefits)
	Socio-economic level and other information (income quintile, disability registration information)
**MT-DB**	Medical institution information
	Medical care benefit costs
	Information on medical subjects and medical diseases
	Details of medical examination, treatment, surgery and other acts, treatment materials, etc.
	Detailed diseases history
	In-house/outpatient prescription drug prescription
**GHE-DB**	Major test results such as body measurement, blood test
	Results of interview about history, lifestyle
	Balance, bone density test, depression, cognitive function test result
**MCI-DB**	Medical utilization, medical institution type and establishment division, medical institution, local information
	Information on the number of beds, doctors, equipment, etc.
**LCI-DB**	Long-term care application and judgment result
	Doctor’s note
	Billing statement
	Basic information on long-term care facilities

**Table 4:** Normal/abnormal criteria of GHE-DB features.

No.	Feature	Class	
		Normal	Abnormal
**1**	Body mass index (kg/m^2^)	0–29	30–300
**2**	Waist circumference (cm)	Male: 50–90, Female: 50–85	Male: 90–130, Female: 85–130
**3**	Blood pressure highest (mmHg)	60–139	140–400
**4**	Blood pressure lowest (mmHg)	40–89	90–250
**5**	Blood glucose before meals (g/dL)	25–125	126–999
**6**	Total cholesterol (mg/dL)	40–229	230–999
**7**	Hemoglobin (g/dL)	Male: 12–16.5, Female: 10–15.5	Male: 0–12, Female: 0–10
**8**	Urine protein	Negative	Positive
**9**	Serum GOT (U/L)	0–50	51–999
**10**	Serum GPT (U/L)	0–45	46–999
**11**	Gamma GTP (U/L)	Male: 11–77, Female: 8–45	Male: 78–999, Female: 46–999

**Table 5:** Known dementia-related diseases.

Disease Classification	Disease
**Degenerative diseases**	(1) Parkinson’s disease, (2) Huntington’s disease, (3) Pick’s disease, (4) Progressive palsy, (5) Multiple system atrophy, (6) Genetic disorder, (7) Motor neuron disease, (8) Multiple sclerosis
**Deficiency disease**	(1) Thiamine (B1): Wernicke’s encephalopathy, (2) Vitamin B12: Pernicious anemia, (3) Nicotinic acid: Pellagra
**Endocrine and organ dysfunction diseases**	(1) Hypothyroidism, (2) Lack of adrenal function and Cushing’s syndrome, (3) Hypothyroidism and hypertrophy, (4) Loss of renal function, (5) Liver failure, (6) Loss of lung function
**Tumor disease**	(1) Primary brain tumor, (2) Paraneoplastic limbic encephalitis, (3) Metastatic brain tumor
**Chronic infection**	(1) Human immunodeficiency virus, (2) Neurosyphilis, (3) Parvovirus, (4) Prion disease, (5) Tuberculosis, (6) Fungi, (7) Protozoa, (8) Sarcoidosis, (9) Whipple’s disease
**Head trauma and extensive brain injury**	(1) Chronic subdural hematoma, (2) Anoxic syndrome, (3) Encephalitis, (4) Normal pressure hydrocephalus
**Toxic disease**	(1) Drug and drug addiction, (2) Alcoholism, (3) Heavy metal poisoning, (4) Organic toxins
**Mental illness**	(1) Depression, (2) Schizophrenia, (3) Conversion reaction
**Other**	(1) Vasculitis, (2) CADASIL, (3) Acute intermittent porphyria, (4) Repeated seizures

**Table 6:** Features of baseline experiment.

Year	DB	Features
**2013**	PIE-DB	(1) Sex, (2) Age, (3) Income quintile
	GHE-DB	(1) Height, (2) Weight, (3) Body mass index, (4) Waist, (5) Blood pressure highest, (6) Blood pressure lowest, (7) Blood sugar before meals, (8) Total cholesterol, (9) Hemoglobin, (10) Urine protein, (11) Serum GOT, (12) Serum GPT, (13) Gamma GTP, (14) History of personal illness: Stroke, Heart disease, High blood pressure, Diabetes, Hyperlipidemia, Phthisis, Cancer, (15) History of family illness: Stroke, Heart disease, High blood pressure, Diabetes, cancer
	MT-DB	Personal disease history diagnosis by every year

**Table 7:** Features of longitudinal data-based experiment set.

DB	Features	
**PIE-DB**		Increasing/decreasing compared to 2013 [Income quintile]
		Class changing compared to 2013 [Income quintile]
**GHE-DB**	Features of baseline	Increasing/decreasing compared to 2013 [(1) Height, (2) Weight, (3) Body mass index, (4) Waist, (5) Blood pressure highest, (6) Blood pressure lowest, (7) Blood sugar before meals, (8) Total cholesterol, (9) Hemoglobin, (10) Urine protein, (11) Serum GOT, (12) Serum GPT, (13) Gamma GTP, (14) History of personal illness: Stroke, Heart disease, High blood pressure, Diabetes, Hyperlipidemia, Phthisis, cancer, (15) History of family illness: Stroke, Heart disease, High blood pressure, Diabetes, Cancer]
		Class changing compared to 2013 [(1) Body mass index, (2) Waist, (3) Blood pressure highest, (4) Blood pressure lowest, (5) Blood sugar before meals, (6) Total cholesterol, (7) Hemoglobin, (8) Urine protein, (9) Serum GOT, (10) Serum GPT, (11) Gamma GTP, (12) History of personal illness: Stroke, Heart disease, High blood pressure, Diabetes, Hyperlipidemia, Phthisis, Cancer, (13) History of family illness: Stroke, Heart disease, High blood pressure, Diabetes, Cancer]
**MT-DB**		Personal disease history diagnosis by every year

**Table 8:** Longitudinal model 1 (All MT-DB features with primary disease group).

	Baseline	Longitudinal Model 1				
**Year**	2013	2003–2013	2005–2013	2007–2013	2009–2013	2011–2013
**Number of features**	55	366	314	262	210	158
**True positive**	614	638	613	630	648	648
**False positive**	317	285	280	282	274	292
**True negative**	533	565	570	568	576	558
**False negative**	236	212	237	220	202	202
**Accuracy (%)**	67.5	70.8	69.6	70.5	72.0	70.9
**Precision (%)**	66.0	69.1	68.6	69.1	70.3	68.9
**Recall (%)**	72.2	75.1	72.1	74.1	76.2	76.2
**F-measure (%)**	69.0	72.0	70.3	71.5	73.1	72.4

**Table 9:** Longitudinal model 2 (Best combination of MT-DB features).

	Baseline	Longitudinal Model 2				
**Year**	2013	2003–2013	2005–2013	2007–2013	2009–2013	2011–2013
**Number of features**	55	709	559	409	259	113
**True positive**	614	623	625	633	619	611
**False positive**	317	78	79	82	69	67
**True negative**	533	772	771	768	781	783
**False negative**	236	227	225	217	231	239
**Accuracy (%)**	67.5	82.1	82.1	82.4	82.4	82.0
**Precision (%)**	66.0	88.9	88.8	88.5	90.0	90.1
**Recall (%)**	72.2	73.3	73.5	74.5	72.8	71.9
**F-measure (%)**	69.0	80.3	80.4	80.9	80.5	80.0

**Table 10:** Best feature combination.

DB	Primary Disease Group	Type	Features
**MT-DB**	E; Endocrine, nutritional and metabolic diseases (8)	Known	(1) Other disorders of pancreatic internal secretion, (2) Vitamin D deficiency, (3) Other disorders of thyroid, (4) Malnutrition-related diabetes mellitus
		Newly detected	(1) Hyperfunction of pituitary gland, (2) Hypofunction and other disorders of pituitary gland, (3) Other disorders of adrenal gland, (4) Unspecified protein-energy malnutrition
	F; Mental and behavioural disorders (13)	Known	(1) Dementia in Alzheimer’s disease, (2) Vascular dementia, (3) Mental and behavioural disorders due to use of alcohol, (4) Acute and transient psychotic disorders, (5) Unspecified nonorganic psychosis, (6) Unspecified dementia, (7) Bipolar affective disorder, (8) Depressive episode, (9) Delirium, not induced by alcohol and other psychoactive substances, (10) Eating disorders, (11) Psychological and behavioural factors associated with disorders or diseases classified elsewhere, (12) Other mental disorders due to brain damage and dysfunction and to physical disease, (13) Schizophrenia
	G; Diseases of the nervous system (17)	Known	(1) Parkinson’s disease, (2) Secondary parkinsonism, (3) Parkinsonism in diseases classified elsewhere, (4) Alzheimer’s disease, (5) Other degenerative diseases of nervous system NEC, (6) Epilepsy, (7) Status epilepticus, (8) Transient cerebral ischaemic attacks and related syndromes, (9) Vascular syndromes of brain in cerebro- vascular diseases, (10) Disorders of other cranial nerves, (11) Hemiplegia, (12) Paraplegia and tetraplegia, (13) Other paralytic syndromes, (14) Hydrocephalus, (15) Other disorders of brain, (16) Other disorders of nervous system, NEC, (17) Other disorders of nervous system in diseases classified elsewhere
	I; Diseases of the circulatory system (7)	Known	(1) Hypertensive renal disease, (2) Subsequent myocardial infarction, (3) Cerebral infarction, (4) Cerebrovascular disorders in diseases classified elsewhere, (5) Sequelae of cerebrovascular disease, (6) Aortic aneurysm and dissection, (7) Stroke, not specified as haemorrhage or infarction
	N; Diseases of the genitourinary system (8)	Known	(1) Acute nephritic syndrome, (2) Chronic kidney disease, (3) Glomerular disorders in diseases classified elsewhere
		Newly detected	(1) Calculus of lower urinary tract, (2) Urethral stricture, (3) Other disorders of male genital organs, (4) Inflammatory disease of uterus, except cervix, (5) Polyp of female genital tract
	M; Diseases of the musculoskeletal system and connective tissue (3)	Newly detected	(1) Kyphosis and lordosis, (2) Spinal osteochondrosis (3) Psoriatic and enteropathic arthropathies
	R; Symptoms, signs and abnormal clinical and laboratory findings, NEC (13)	Known	(1) Faecal incontinence, (2) Abnormalities of gait and mobility, (3) Unspecified urinary incontinence, (4) Somnolence, stupor and coma, (5) Other symptoms and signs involving cognitive functions and awareness, (6) Other symptoms and signs involving general sensations and perceptions, (7) Symptoms and signs involving appearance and behavior,
		Newly detected	(1) Ascites, (2) Retention of urine, (3) Voice disturbances, (4) Malaise and fatigue, (5) Enlarged lymph nodes, (6) Systemic Inflammatory Response Syndrome
	S; Injury, poisoning and certain other consequences of external causes (6)	Known	(1) Fracture of skull and facial bones, (2) Open wound of thorax, (3) Injury of other and unspecified intrathoracic organs, (4) Open wound of forearm, (5) Fracture at wrist and hand level, (6) Injury of muscle and tendon at hip and thigh level
**GHE-DB**	-	-	(1) Total cholesterol, (2) Hemoglobin, (3) Serum GOT, (4) Serum GPT, (5) Gamma GTP
